# The Wechsler intelligence scale for children, fourth and fifth editions perform comparably in children with Batten disease

**DOI:** 10.1186/s13023-025-03923-w

**Published:** 2025-08-07

**Authors:** Heather R. Adams, Erika F. Augustine, Kristen Bonifacio, Alyssa Collins, Amy E. Vierhile, Jonathan W. Mink

**Affiliations:** 1 Department of Neurology, Division of Child Neurology, University of Rochester Medical Center, 601 Elmwood Avenue Box 631, Rochester, NY 14642 USA; 2 Clinical Trials Unit, Kennedy Krieger Institute, 707 North Broadway, Baltimore, MD 21205 USA; 3 New York State Department of Health, AIDS Institute, Albany, NY 12220 USA; 4 Child Life Program, Golisano Children’s Hospital, University of Rochester Medical Center, 601 Elmwood Avenue, Rochester, NY 14642 USA; 5 Consultant, Pittsford, NY 14534 USA

**Keywords:** Batten disease, Neuronal ceroid lipofuscinoses, Cognition, Wechsler scales, Convergent validity

## Abstract

**Background:**

The neuronal ceroid lipofuscinoses (Batten disease) are rare neurodegenerative lysosomal storage diseases principally of childhood onset and an autosomal recessive inheritance pattern. Cognitive regression is a hallmark of the disease, and has been characterized as part of the University of Rochester Batten Center’s prospective longitudinal natural history. The objective of the present study was to establish convergent validity of the two most recent versions of the Wechsler Intelligence Scale for Children in this population (WISC-IV, 2003; WISC-V, 2014) due to anticipated eventual obsolescence of WISC-IV. 18 children and young adults (12 males, 6 females) with a genetically confirmed NCL diagnosis were administered selected subtests from the WISC-IV and WISC-V. We used bivariate correlations and repeated measures ANOVA between matching subtests across these two WISC versions to determine convergence of the measures.

**Results:**

WISC-IV and WISC-V verbal subtests were strongly correlated with one another and mean age-adjusted scores for comparable subtests on WISC-IV vs. WISC-V were not significantly different from one another.

**Conclusions:**

Overall, the minimal performance differences on the two measures supports combining WISC-IV and WISC-V datasets for larger-scale analyses of the neurocognitive natural history of NCL disorders.

## Background

The NCLs are a set of primarily pediatric-onset, inherited lysosomal storage diseases resulting from variants in one of 13 distinct genes [1]. With the exception of cerliponase alpha for treatment of CLN2 disease [2]there are no currently approved disease-modifying therapies available for the NCLs. The various genetic forms of the NCLs are clinically differentiated from one another to some extent by age of onset, rate of disease progression and temporal order of symptom onset [3, 4]. Despite these differences, most NCL disorders are characterized by cognitive and developmental regression, vision loss, loss of motor and language abilities, seizures, and premature death [3, 5, 6]. Longitudinal, natural history studies have thus far mapped the natural history of cognition in two NCL types - CLN2 [7, 8] and CLN3 [9–12] disease. Though the individual course may vary, among children with the classic late-infantile CLN2 phenotype, delays in attaining cognitive and language milestones are evident as early as 24 months of age, followed by a rapid loss of abilities over an approximately 2–3 year period. Children with CLN3 disease typically exhibit normal cognitive development in their early years but most reach a plateau in skills between approximately 10–15 years of age and then experience cognitive decline and loss of adaptive skills [13]. Numerous case reports and case series of other NCL diseases have also similarly described cognitive decline after either a period of typical or delayed attainment of cognitive developmental milestones in early childhood [14].

Because some NCL disorders, such as classic CLN3 disease are slowly progressing over several decades, long-range natural history studies are needed to completely characterize the clinical phenotype, timing of symptom onset, and rate of symptom progression. Over approximately two decades, the University of Rochester Batten Center (URBC) has used subtests from the Wechsler Intelligence Scale for Children (WISC) as one component of a prospective, longitudinal natural history study to characterize neurobehavioral disease progression in the NCLs [15, 16]. In 2004 when our center began using the fourth edition of the WISC (WISC-IV) to assess cognition in NCL disorders, it was a newly validated instrument (2003) with updated materials and normative data that were well-matched to the generational cohort under study at the time. In a planned update, the test publisher (Pearson) released the Wechsler Intelligence Scales for Children– Fifth Edition (WISC-V) in 2014. Periodic revisions and updates to cognitive assessments are necessary to adjust for the ‘Flynn effect’, the phenomenon of increasing IQ scores over time, causing earlier normative data to become outdated and necessitating re-standardization of test norms [17]. Additionally, some test stimuli may become obsolete with changes in technology and culture.

Like its predecessor, the WISC-V is validated for use in children age 6–16 years old [18, 19]. Revisions to the WISC from the fourth to fifth edition included changes to item content and scoring (e.g., the total number of items included per subtest impacted subtest raw score totals), the addition of several new primary and supplementary subtests, and reorganization of the test factor structure [20]. The WISC-V also established an updated normative sample stratified to be representative of key sociodemographic variables in the United States (race, ethnicity, sex, parental education, geographic region) [20]. Convergent validity between the WISC-IV and WISC-V was established in a general population sample by administering the two tests to children in a counterbalanced order and examining correlation coefficients and mean score differences between corresponding index scores and subtests; overall there were moderate to high relationships between analog subtests across the two test versions. While most composite index (second-order factor) scores were lower on the newest version of the test the differences were of a small magnitude and not deemed to be clinically relevant (e.g., Verbal Comprehension Index, etc.) [20, 21]. Test-criterion validity of the WISC-V was also evaluated in small subsamples of particular clinical groups of interest (e.g., Attention-Deficit/Hyperactivity Disorder [ADHD], autism spectrum disorder, intellectual disability and learning disability). However, convergent validity between WISC-IV and WISC-V was not evaluated in these special group sub-studies. In addition, though the updating and revalidation of the WISC from version IV to V included several common clinical subpopulations, this work did not include individuals with rare or neurodegenerative disorders. Thus, it was unknown if the two versions (WISC-IV, WISC-V) would be comparable for children with an NCL disorder, whose unique clinical picture distinguishes them from the type of children included in the WISC-V standardization sample. However, it is important to differentiate between changes in test scores that are related to disease rather than to test revisions over time. Sensitivity to this concern was addressed in consensus recommendations generated to assess neurocognition in clinical trials for another set of neuronopathic lysosomal storage disorders, the mucopolysaccharidoses (MPS), including considerations for “… consistency and comparability to past data collected” (p. 187) [22]. Therefore, anticipating eventual obsolescence of the WISC-IV and hence a need to transition to the WISC-V for ongoing natural history work, we compared analogous WISC-IV and WISC-V subtests. Our goal was to ascertain convergent validity between these measures in order to determine continued relevance of the newest WISC version for our population of interest and whether WISC-IV and WISC-V data could be combined for future analyses.

## Methods

### Participants

Children and adolescents with a genetically confirmed NCL diagnosis participated in this study. Affected individuals were recruited and evaluated during visits by them and their parents/guardians to the University of Rochester Batten Center (URBC, Rochester, NY), or at annual family meetings of the Batten Disease Support, Research, and Advocacy Foundation (BDSRA) Foundation. Genetic diagnosis was confirmed by review of clinical records provided by the family or through direct testing at our center.

The research was conducted under a study protocol approved by the University of Rochester Research Subjects Review Board (RSRB Study00001970). Parents / legal guardians provided written permission for the participation of the affected individual; the requirement to obtain assent (from children) and informed consent (from adults) with Batten disease was waived by the RSRB due to lack of capacity resulting from the cognitive impairments of the affected individuals.

### Neuropsychological assessment

Full details of the URBC’s approach to cognitive assessment of children with NCL disorders have been published [15, 23]. For the current project, conducted between 2017 and 2018, we co-administered selected subtests present in both the WISC-IV and WISC-V core batteries. Because of the cognitive limitations of participants, ceiling effects were not a concern and therefore it was possible to consistently administer a version of the child-age test (i.e., WISC) irrespective of chronological age. To accommodate vision loss, only verbally-mediated tasks were administered, in particular, tasks to assess verbal reasoning (Similarities subtest), vocabulary (Vocabulary subtest), fund of knowledge (Information), and auditory attention and working memory (Digit Span subtest). Of note, the WISC-IV Digit Span subtest contains two sub-tasks, “Digit Span Forward” which involves verbatim repetition of number sequences (DSF) and “Digit Span Backward” which involves repetition of sequences in reverse order from their presentation (DSB). The WISC-V revision adds a third element, “Digit Span Sequencing” requiring examinees to re-organize the number spans in sequential order. On each sub-task, the sequence length increases as the test progresses. Because our major question was to determine convergence of the same tests across the two WISC versions, this project focused upon comparing the respective performance on the two individual sub-tasks included in both WISC versions (DSF, DSB).

Evaluations were conducted in-person at annual family meetings of the BDSRA Foundation and at the URBC. Both WISC versions were presented during the same testing session; the order of test version administration was counterbalanced across subjects. Due to longitudinal participation in our studies, some children participated in completing both assessments in more than one year of the project. Where overlap of item stimuli existed across test versions for the Vocabulary, Similarities, and Information subtests, within-session practice effects were minimized by administering overlapping items (i.e., same item appearing in each test version) only one time, that is, within the test that was administered first. Despite such items being administered only once, they were nonetheless scored separately for WISC versions IV and V according to the specific scoring rubrics for the respective test. For the Similarities subtest, the total item number (23 items) was the same for each test version. Across the two versions, 30% (7/23) of items overlapped exactly (verbatim) and were located in an identical position (or only one position before or after) within the test. Another 30% (7/23) of items had overlapping content (e.g., comparison of two different types of animals) and the same or similar sequence (within 1–2 positions) in the test, i.e., item difficulty level was considered similar across test versions. In the Vocabulary subtest, the total item count was reduced from 36 (WISC-IV) to 29 (WISC-V) items. For this subtest, the number of overlapping items reflected 42% of the WISC-IV (15/36) and 52% of the WISC-V (15/29) version. Item difficulty appeared similar based on the relative positions of the overlapping items. Because the majority of stimuli for both versions of Digit Span (over 85%) and for the Information subtest (over 70%) were different, each WISC version of these subtests were administered in their entirety to each subject.

### Statistical analysis

Subjects ≥ 17 years old were assigned age-standardized scores for children 16 years, 11 months old, the maximum age for both WISC-IV and WISC-V normative test samples. Statistica version 13 [24] was used to conduct statistical analyses. We evaluated pairwise, bivariate correlations between matching subtests (e.g., WISC-IV vs. WISC-V Similarities) and used intraclass correlation (ICC) to measure agreement between subjects’ pairs of test scores across matching subtests. To evaluate differences between mean scaled (i.e., age-normed) scores on the same tests for WISC-IV vs. WISC-V versions and control for multiple comparisons, we performed one-way repeated-measures analysis of variance (ANOVA) tests, with “test version” (WISC-IV vs. WISC-V) as a 2-level categorical factor.

## Results

18 subjects (12 males, 6 females) participated in the study. The sample included individuals with the following NCL disorders: CLN1, *n* = 1; CLN3, *n* = 14; and CLN6, *n* = 3. Average age when first assessed was 13.8 years (standard deviation, = 4.9; range = 6.4–21.0 years).

Due to the longitudinal nature of the URBC natural history study, 3 subjects participated in more than one study year. This resulted in a total of 21 assessments with at least two or more matching subtests from each WISC version being administered (e.g., WISC-IV *and* WISC-V Similarities subtest). Time constraints on study visits or the impact of dementia symptoms limited our ability to complete all matching subtests in each assessment. Of the 21 assessments completed, individual subtest completion for both versions of the WISC was: Similarities, *n* = 18; Information, *n* = 17; Vocabulary, *n* = 19; Digit Span Forward, *n* = 19; Digit Span Backward, *n* = 11. Even among subjects who were ≥ 17 years old, ceiling effects were not a concern due to cognitive regression; no subject obtained the maximum raw score on any subtest.

**Fig. 1:**
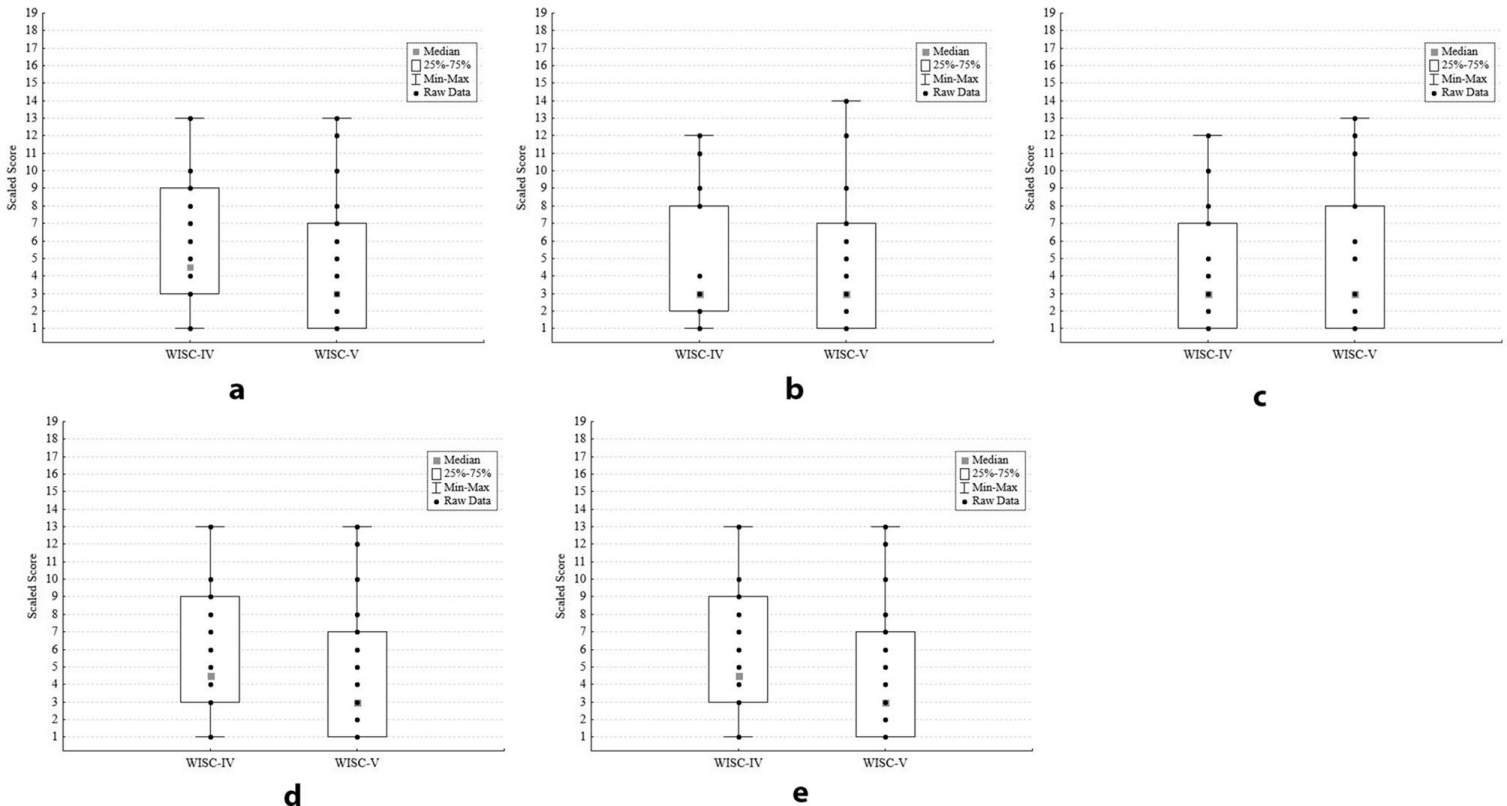
(**a**) Similarities Subtest: Distribution of WISC-IV and WISC-V Scaled Scores. (**b**) Information Subtest: Distribution of WISC-IV and WISC-V Scaled Scores. (**c**) Vocabulary Subtest: Distribution of WISC-IV and WISC-V Scaled Scores. (**d**) Digit Span Forward Subtest: Distribution of WISC-IV and WISC-V Scaled Scores (**e**) Digit Span Backward Subtest: Distribution of WISC-IV and WISC-V Scaled Scores. *Description*: For each figure (**a**-**e**), boxplots and whiskers present median, 25th -75th percentile and minimum and maximum values. Individual participants’ raw scores on WISC-IV and WISC-V subtests are shown within each boxplot. There are no outlier values

**Fig. 2:**
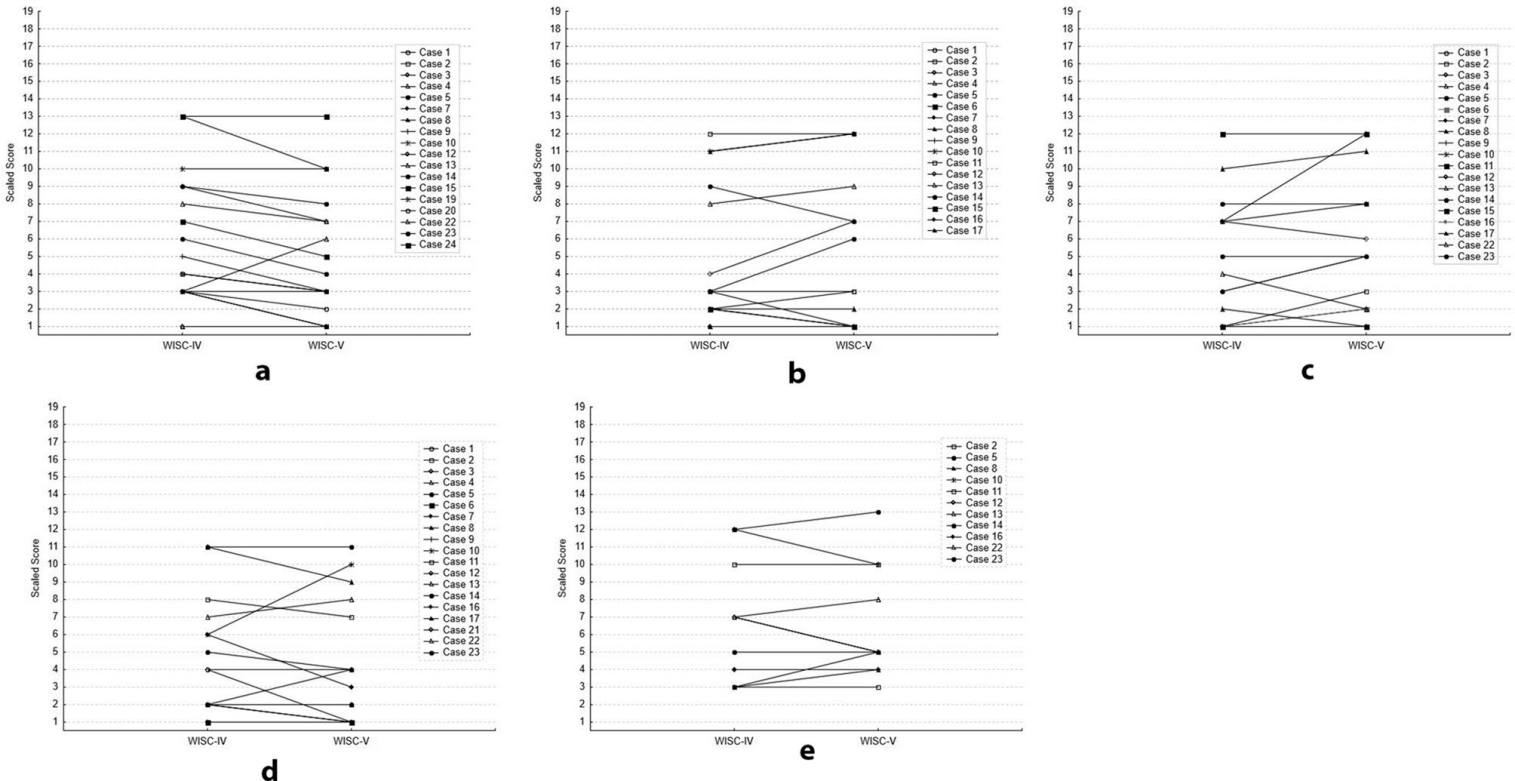
(**a**) Similarities Subtest (**b**) Information Subtest (**c**) Vocabulary Subtest (**d**) Digit Span Forward Subtest (**e**) Digit Span Backward Subtest *Description*: Each figure (**a**-**e**) presents individual (paired) WISC-IV and WISC-V data on each subtest, to illustrate within-subject change in subtest scaled scores

Figure 1 (a-e) presents box plots that show the distribution of scores for each WISC-IV vs. WISC-V subtest comparison. WISC-IV and WISC-V subtests were highly correlated for tasks of abstract reasoning (WISC-IV. vs. WISC-V *Similarities*, *r* =.94, *p* <.001); fund of knowledge (*Information*, *r* =.95, *p* <.001), vocabulary skills (*Vocabulary*, *r* =.92, *p* <.001), attention (*Digit Span Forward*, *r* =.89; *p* <.001), and working memory (*Digit Span Backward*, *r* =.92, *p* <.001). In only *n* = 11 of the assessments was the Digit Span Backward task completed due to the task’s difficulty for subjects with greater cognitive impairment. Figure 2**(**2a-2e) presents individual (paired) WISC-IV and WISC-V data on each subtest, to illustrate the minimal within-subject change in subtest scaled scores.

Also, as noted above, one-way repeated measures ANOVA analyses were used to compare mean scaled scores on the same WISC-IV and WISC-V subtests. There were no significant differences between WISC-IV and WISC-V subtest mean scores based on this analysis; all tests were two-sided: Similarities subtest WISC-IV mean (s.d.) = 5.83 (3.76), WISC-V mean (s.d.) = 4.89 (3.61): *F*_1,17_=2.06, *p* =.17; Information subtest WISC-IV mean (s.d.) = 4.47 (3.99), WISC-V mean (s.d.) = 4.71 (4.33): *F*_1,17_=0.00, *p* =.98; Vocabulary subtest, WISC-IV mean (s.d.) = 4.00 (3.51), WISC-V mean (s.d.) = 4.58 (3.92): *F*_1,17_=0.08, *p* =.78; Digit Span Forward subtest, WISC-IV mean (s.d.) = 4.05 (3.31), WISC-V mean (s.d.) = 3.79 (3.46): *F*_1,17_=0.17, *p* = 0. 88; Digit Span Backward subtest, mean (s.d.) = 7.08 (3.63), WISC-V mean (s.d.) = 6.55 (3.21): *F*_1,17_=0.06, *p* =.81. Intraclass correlation (ICC) estimates were also calculated to assess agreement between the test version scores. Each of these was non-significant: Similarities subtest ICC = 0.10; Information subtest ICC= -0.12; Vocabulary subtest ICC= -0.12; Digit Span Forward subtest ICC= -0.10; Digit Span Backward subtest ICC= -0.11.

To assess comparability of an overall estimate of verbal reasoning skills, we also compared scores on a “Verbal Composite” which we calculated as the mean scaled score of three subtests: Similarities, Vocabulary, Information. This was available for *n* = 17 subjects; all but one had a CLN3 disease diagnosis; one other individual had CLN1 disease. As expected, based on the absence of significant differences between individual subtest comparisons, there was similarly no significant difference between the WISC-IV and WISC-V Verbal composite (WISC-IV Mean = 4.75, SD = 3.76; WISC-V Mean = 4.73, SD = 3.87; *t* = 0.11, df = 16; 95% CI: -0.41, 0.38).

## Discussion

The NCLs are rare and predominantly pediatric-onset inherited neurodegenerative diseases. Despite clinical differences across and within the various genetically distinct forms of NCL disorders with respect to patterns of symptom onset and severity, rate of disease progression and duration of disease, cognitive decline is a common theme across all NCL types. In the current study convergent validity was established for analogous WISC-IV and WISC-V subtests in individuals with NCL disorders. The small sample size was an anticipated limitation, in light of the focus on this set of rare diseases. Practical considerations limited the ability to conduct WISC-IV and WISC-V assessments at separate testing sessions which is a typical approach for evaluating convergence between two similar measures to reduce practice effects. We attempted to mitigate this risk by limiting exposure to identical items across the two versions of the WISC though acknowledge that it resulted in a somewhat modified testing approach. We also counterbalanced the order of assessments to reduce systematic influence of one test version upon performance on the other. Of note, it was not possible to establish convergent validity across all subtests of the two WISC versions, given that participants’ vision loss precluded administration of the visually-based subtests that assess domains of nonverbal and fluid reasoning, visual-spatial processing, and processing speed.

By definition, rare diseases involve small groups of individuals, presenting research challenges including slow accrual of data to acquire information from a sufficient number of patients to inform for study design and data analysis [25]. This challenge is further amplified in diseases with slowly progressing symptoms that require extended follow-up periods to establish the trajectory of symptom changes over time. Longitudinal natural history studies help establish a sufficient evidence base on disease phenomenology and the patient experience, identify targets and critical time points for intervention, and establish reliable and meaningful clinical outcome measures. In addition, the United States Food and Drug Administration (FDA) has also acknowledged conditions under which natural history data could itself serve as an external control group in a clinical trial [26]. Understanding the cognitive phenotype of neuronopathic inherited diseases is critical for several reasons. Cognitive symptoms and their change over time may provide insights into underlying disease pathology, support phenotype differentiation, and may serve as an indicator of disease progression [27]. Further, accurate and precise measurement of disease-related cognitive change over time may guide estimation of a clinically meaningful alteration in its trajectory in response to an experimental therapy. Finally, even in the absence of disease-modifying therapies, knowledge of a disease’s associated cognitive phenotype and natural history enables better targeted support and anticipatory guidance for affected patients and families.

The long duration of our prospective longitudinal study now spans two versions of the Wechsler Intelligence Scale for Children, which undergoes updates and revisions approximately every one to two decades to remain a contemporary measure of intellectual ability. The importance of establishing concordance between older and newer versions of the same test is routinely addressed when test publishers introduce test revisions, as part of the psychometric validation process. To our knowledge there are no published studies that establish convergent validity between different versions of the same cognitive assessment for the evaluation of children with neuronopathic diseases. However, a related concern has been addressed for these populations, which is the transition between tests of cognition because of increasing age (chronological or developmental) over the course of a study (e.g., from the preschool- to school-age version of the Wechsler IQ test). A similar methodologic approach is recommended as the one we have taken, i.e., to overlap the tests by administering both at the same time point in order to determine comparability of the findings [28].

## Conclusion

With the release of the WISC-V during our ongoing study which began with use of the WISC-IV, it was necessary to determine if continuity could be maintained in our approach to measuring cognition in this rare disease despite transitioning to an updated WISC test version. Overall, the results suggest continuity in measurement for individuals evaluated by both measures, enabling WISC-IV and WISC-V data collected by the URBC to be combined, ensuring the largest possible dataset for analysis within diagnostic subtype for each of these respective rare disorders. A similar approach could be undertaken in other specialized populations when there is a need to transition from older to a more contemporary version of the same assessment.

## Data Availability

The datasets generated and/or analysed during the current study are not publicly available due to concern about identifiability of individuals with these ultrarare diseases; deidentified may be available from the corresponding author on reasonable request and in compliance with institutional policies for data sharing.

## Abbreviations

ADHD: Attention-Deficit/Hyperactivity Disorder
ANOVA: Analysis of Variance
BDSRA: Batten Disease Support, Research, and Advocacy Foundation
DSF: Digit Span Forward
DSB: Digit Span Backward
FDA: United States Food and Drug Administration
ICC: Intra-class correlation
MPS: mucopolysaccharidoses
NCL: Neuronal Ceroid Lipofuscinosis
RSRB: Research Subjects Review Board
s.d: standard deviation
URBC: University of Rochester Batten Center
WISC-IV: Wechsler Intelligence Scale for Children, Fourth edition
WISC-V: Wechsler Intelligence Scale for Children, Fifth edition
